# Supplementary material for: Heuristic assessment of choices for risk network control
Source: Sci Rep. 2021 Apr 7;11:7645. doi: 10.1038/s41598-021-85432-x (PMC8026632; doi:10.1038/s41598-021-85432-x)
Supplement: Supplementary file 1 — Supplementary Information 1. [file 41598_2021_85432_MOESM1_ESM.pdf]

Supplementary Information for

Heuristic assessment of choices for risk network control

Xiang Niu, Christopher Brissette Chunheng Jiang, Jianxi Gao, Gyorgy Korniss, Boleslaw K. Szymanski

Boleslaw K. Szymanski
E-mail: szymab@rpi.edu

This PDF file includes:

Supplementary text
Figs. S1 to S5
References for SI reference citations

Supporting Information Text

Global Risk Network Model

According to the World Economic Forum (WEF) experts, a global risk is defined as "an uncertain event or condition that, if it occurs, can cause a significant negative impact for several countries or industries within the next ten years" (1). Experts also categorize the global risks, measure the mutual relationships and analyze their likelihoods and impacts annually. The global risks interconnections are extracted from a survey with participants' answer to the question: "Global risks are not isolated and it is important to assess their interconnections. In your view, which are the most strongly connected global risks? Please select three to six pairs of global risks" . In this way, the interconnection between any pair of risks is weighted by the number of respondents. For simplicity, in our paper, we formulate unweighted undirected global risk network E without considering the weights of the risks interconnections, where $E_{ij} = 1, i \neq j$ indicates that the risk i and j are thought as one of most strongly connected global risks from at least one respondent. For the rest of risk pairs (i, j), $E_{i,j} = 0$. For each risk i, the likelihood l_i is averaged over the participants' answers to the question "What is the likelihood of it occurring globally within the next 10 years?" ; the impact represents "What is the negative impact for several countries or industries within the next ten years?" . The likelihood is measured on a scale of 1–7 and the impact on a scale of 1–5 . In our paper, we use risks likelihoods to establish the Cascading Alternating Renewal Processes (CARP) model to simulate global risk cascading over the years, while leaving the risk impact with no discussion. However, it is also an important factor in global risks analysis and may bring up another topic in future research.

Over the years global risk reports in WEF, the five risk categories remain unchanged, including "Economic", "Environmental", "Geopolitical", "Societal", and "Technological". However, the risks coevolve with their networks, likelihoods, and the impacts. From Jan 2000 to Dec 2016, there are 17×12 monthly data points in total for each different risk. Each data point is determined by human knowledge from online event resources such as articles, news, Wikipedia, etc (2). At a specific month k, the state $x_i(k)$ of risk i is labeled active (1) or inactive (0). Fig. S1 shows the 17×12 months historical data for 30 risks in 2017 global risk network, which is consistent with the data used in (2).

The Cascading Alternating Renewal Processes (CARP) was proposed to analyze global risk cascading by researchers in (2). In the process, the state of a risk is either active (1) or dominant (0). During each step k, the states of risks are updated simultaneously with the following three transitions:

- Internal activation: an inactive risk i is activated internally with probability p_i^{int}.
- External activation: an inactive risk i is activated externally by an active node j with probability $p_{ji}^{ext} = p_i^{ext}$.
- Internal recovery: an active risk i is inactivated internally with probability $p_i^{rec} = 1 - p_i^{con}$.

The internal activation and recovery are further combined as endogenous dynamic $F[\vec{x}(k)] = \vec{p}^{int} \circ [1 - \vec{x}(k)] + \vec{p}^{con} \circ \vec{x}(k)$ of the system, while the external activation forms exogenous dynamic $G[\vec{x}(k), E] = [E^{\intercal}\vec{x}(k)] \circ \vec{p}^{ext} \circ [1 - \vec{x}(k)]$.

To get the reliable probabilities p_i^{int}, p_i^{ext} and p_i^{rec} that mostly reflect the unique activation likelihood and the consistent activation intensity of risk i, the researchers further introduce intensity parameters of internal activation α, of external activation β, and of internal recovery γ (2). With normalized likelihood L_i in Fig. S1 and intensity parameters, the activation probabilities of risk i are: $p_i^{int} = 1 - (1 - L_i)^{\alpha}$, $p_i^{ext} = 1 - (1 - L_i)^{\beta}$, $p_i^{con} = 1 - (1 - L_i)^{\gamma}$. In a probabilistic model where $x_i(k) \in [0, 1]$ is the frequency of a risk i being active at step k, the system discrete state transition function is

$$\vec{x}(k+1) = F(\vec{x}(k)) + G(\vec{x}(k), E). \quad [1]$$

However, in a deterministic model where $x_i(k) \in \{0, 1\}$ is the deterministic state of a risk i being active at step k in a simulation or historical record,

$$x_i(k+1) = \begin{cases} 1, \text{with probability } p_i(k), \\ 0, \text{with probability } 1 - p_i(k), \end{cases} \quad [2]$$

where state transition probability $\vec{p}(k) = F(\vec{x}(k)) + G(\vec{x}(k), E)$, $p_i(k)$ is the ith value of vector $\vec{p}(k)$. Given historical deterministic state $x_i(k)$ of risk i at monthly step k, it is easy to get state transition probability $p_i(k)$ of risk i at month k, and the product of state transition probabilities $L_p = \prod_{k=0}^{\tau-1} \prod_{i=1}^{N} p_i(k)$ over the integrated system with N risks and entire period of historical events with $\tau = 17 \times 12$ months. By maximizing log-likelihood $\ln L_p$, we get the optimal intensity parameters α, β and γ from the 2013 to 2017 global risk networks in Table 1.

As discussed in (2), the risk parameters α, β, γ are used to map experts' assessments of risk likelihoods of state transition into probabilities. These assessments are remarkably precise, differing from linear mapping by just about 4% based on the Maximum Likelihood Estimation of the transition probabilities from historical data. Hence, the global risk networks based on these assessments are in close agreement with historical transitions of risks between their states. It should also be noted that both risks themselves and their probabilities of transitions between states constantly change; new risks arise and are added to the network, while existing active risks either continue to be a threat and remain in the network but with modified parameters, or, thanks to the response of threatened governments and industry, decline in importance and are removed. This evolution causes continuous changes in the global risks and their probabilities, and leads to the need for annual revisions of the list of risks present in the network and their parameters by the experts. However, if left unabated, the global risk network would approach the steady state, in which some risks will be active much more frequently making their threats much more pronounced than in the initial state.

Optimal Control

In a discrete-time linear system, at time step $k \in \{0, 1, ..., \tau\}$, the state variables are represented by a N dimensional vector $\vec{x}(k) \in \mathbb{R}^{N\times 1}$, the control input variables are represented by a N_D dimensional vector $\vec{u}(k) \in \mathbb{R}^{N_D\times 1}$, τ is the final time step. $x_i(k)$ is the state of node i at time k, $u_j(k)$ is the amount of input on driver node j at time step k. The discrete-time linear system control is

$$\vec{x}(k+1) = A\vec{x}(k) + B\vec{u}(k). \quad [3]$$

In the real world, most network dynamics are much more complicated and cannot be linearly represented. Thus, we need a more general representation of the nonlinear system. In a discrete-time system,

$$\vec{x}(k+1) = f[\vec{x}(k), \vec{u}(k)] = f_x[\vec{x}(k)] + B\vec{u}(k), \quad [4]$$

where f and f_x are N dimensional vector functions, $f : (\mathbb{R}^{N\times 1}, \mathbb{R}^{N_D\times 1}) \to \mathbb{R}^{N\times 1}$, $f_x : (\mathbb{R}^{N\times 1}) \to \mathbb{R}^{N\times 1}$. Specifically, in CARP model,

$$\vec{x}(k+1) = F[\vec{x}(k)] + G[\vec{x}(k), E] + B\vec{u}(k), \quad [5]$$

where F and G are parts of f_x. At some time, the nonlinear dynamics are too difficult to solve directly, thus we may need an approximated but easier and faster linear solution. This approximation is known as the linearization method.

In a discrete-time system, first we choose an operating point $(\vec{x}_s, \vec{u}_s)$ at time k_s, such that $\vec{x}_s = \vec{x}(k_s), \vec{u}_s = \vec{u}(k_s), \vec{x}(k_s+1) = f[\vec{x}(k_s), \vec{u}(k_s)]$. Assume that the trajectory of the nonlinear system is in the neighborhood of the operating point, $\vec{x}(k) = \vec{x_s} + \Delta\vec{x}(k), \vec{u}(k) = \vec{u}_s + \Delta\vec{u}(k)$. We have

$$\begin{aligned} & \vec{x}_s + \Delta\vec{x}(k+1) \\ = \;& \vec{x}(k+1) = f[\vec{x}_s + \Delta\vec{x}(k), \vec{u}_s + \Delta\vec{u}(k)] \\ = \;& f(\vec{x}_s, \vec{u}_s) + \frac{\partial f}{\partial \vec{x}}\Big|_{k=k_s} \Delta\vec{x}(k) + \frac{\partial f}{\partial \vec{u}}\Big|_{k=k_s} \Delta\vec{u}(k) + H.O.T. \end{aligned} \quad [6]$$

where $H.O.T.$ is the higher order terms in the Taylor expansion of $f[\vec{x}(k), \vec{u}(k)]$ (3), and

$$\begin{aligned} \frac{\partial f}{\partial \vec{x}}\Big|_{k=k_s} &= \frac{\partial f_x}{\partial \vec{x}}\Big|_{k=k_s} = \begin{bmatrix} \frac{\partial f_{x1}}{\partial x_1}\Big|_{k=k_s} & \cdots & \frac{\partial f_{x1}}{\partial x_n}\Big|_{k=k_s} \\ \vdots & \ddots & \vdots \\ \frac{\partial f_{xn}}{\partial x_1}\Big|_{k=k_s} & \cdots & \frac{\partial f_{xn}}{\partial x_n}\Big|_{k=k_s} \end{bmatrix}, \\ \frac{\partial f}{\partial \vec{u}}\Big|_{k=k_s} &= B. \end{aligned} \quad [7]$$

Ignoring the high order terms in the Taylor expansion, Eq. (6) becomes

$$\begin{aligned} \Delta\vec{x}(k+1) &\approx \frac{\partial f_x}{\partial \vec{x}}\Big|_{k=k_s} \Delta\vec{x}(k) + B\Delta\vec{u}(k) \\ &= A\Delta\vec{x}(k) + B\Delta\vec{u}(k). \end{aligned} \quad [8]$$

Since with different operating point $(\vec{x_s}, \vec{u}_s)$, the results of linear approximation are different. The most precise approximation always occurs when the selected operating point is an equilibrium point. The equilibrium point is a state that satisfies $\vec{x}(k_s) = \vec{x}(k_s+1), \vec{u}(k_s) = \vec{u}(k_s+1)$ in discrete-time system.

In a discrete-time system, the objective function is

$$J = \sum_{k=0}^{\tau} J_k(\vec{x}(k), \vec{u}(k)). \quad [9]$$

With different functions $J_k : (\mathbb{R}^{N\times 1}, \mathbb{R}^{N_D\times 1}) \to \mathbb{R}^{1\times 1}$, we can achieve different optimal goals J. For example, let $J_k(\vec{x}(k), \vec{u}(k)) = 1$, the optimal goal is to find optimal time length. In most of network control analysis, researchers concern the optimal input cost or the optimal energy used to control the system, then we have

$$J_k(\vec{x}(k), \vec{u}(k)) = \vec{u}^{\intercal}(k)R\vec{u}(k), \quad [10]$$

where $R \in \mathbb{R}^{N_D\times N_D}$ is a cost matrix. In the optimal energy control, R is an identity matrix.

Now our problem can be formulated as finding optimal J under constrains $\vec{x}(k+1) = f(\vec{x}(k), \vec{u}(k))$. To solve this problem, we introduce Lagrangian multipliers $\vec{\lambda}(k) \in \mathbb{R}^{N\times 1}$ and Hamilton function $H : (\mathbb{R}^{N\times 1}, \mathbb{R}^{N_D\times 1}, \mathbb{R}^{N\times 1}, \mathbb{R}^{1\times 1}) \to \mathbb{R}^{1\times 1}$. In a discrete-time system (4)

$$\begin{aligned} H(\vec{x}(k), \vec{u}(k), \vec{\lambda}(k+1)) &= \tfrac{1}{2}J_k(\vec{x}(k), \vec{u}(k)) + \vec{\lambda}^{\intercal}(k+1)f(\vec{x}(k), \vec{u}(k)), \\ \vec{\lambda}(k) &= \tfrac{\partial H(\vec{x}(k), \vec{u}(k), \vec{\lambda}(k+1))}{\partial \vec{x}(k)}, \\ 0 &= \tfrac{\partial H(\vec{x}(k), \vec{u}(k), \vec{\lambda}(k+1))}{\partial \vec{u}(k)}, \\ \vec{x}(k+1) &= \tfrac{\partial H(\vec{x}(k), \vec{u}(k), \vec{\lambda}(k+1))}{\partial \vec{\lambda}(k+1)}. \end{aligned} \quad [11]$$

The previous equations are based on a sufficient condition that $\frac{\partial^2 H}{\partial \vec{u}^2}$ is positive definite. With optimal input cost function $J_k = \vec{u}^\intercal R\vec{u}$ and linear control inputs $f = f_x + B\vec{u}$, $\frac{\partial^2 H}{\partial \vec{u}^2} = R$. For optimal energy control, R is an identity matrix which is positive definite.

To simplify the CARP nonlinear equations, we apply the linearization technique, and get the partial derivative of f_x with respect to $\vec{x}$ at operating point k_s

$$\begin{aligned} \Delta\vec{x}(k+1) &= A\Delta\vec{x}(k) + B\Delta\vec{u}(k), \\ A &= \begin{bmatrix} p_1^{con} - (p_1^{int} + p_1^{ext} E_{1\bullet}^\intercal \vec{x}_s) & \dots & p_1^{ext} E_{1n}^\intercal (1 - x_{s1}) \\ \vdots & \ddots & \vdots \\ p_n^{ext} E_{n1}^\intercal (1 - x_{sn}) & \dots & p_n^{con} - (p_n^{int} + p_n^{ext} E_{n\bullet}^\intercal \vec{x}_s) \end{bmatrix}, \end{aligned} \quad [12]$$

where x_{si} is the ith value of vector $\vec{x}_s$, $\Delta\vec{x}(k) = \vec{x}(k) - \vec{x}_s$, $\Delta\vec{u}(k) = \vec{u}(k) - \vec{u}_s$. Considering there are multiple equilibrium points for any nonlinear risk dynamics, to make all systems comparable with different driver nodes, we chose the equilibrium at $\vec{x}_s = \vec{0}, \vec{u}_s = \vec{0}$.

The variables in Eq. (8) are $\Delta\vec{x}, \Delta\vec{u}$, however, our research targets are the variables of $\vec{x}, \vec{u}$. First, let $\vec{x'}(k) = (A - I)^{-1}(-B\vec{u}_s) + \Delta\vec{x}(k)$, we have

$$\vec{x'}(k+1) = A\vec{x'}(k) + B\vec{u}(k). \quad [13]$$

Thus, the initial states at time 0 are changed to $\vec{x'}(0) = \vec{x'_0} = (A - I)^{-1}(-B\vec{u}_s) + \vec{x_0} - \vec{x_s}$, the final states at time τ are changed to $\vec{x'}(\tau) = \vec{x'_f} = (A - I)^{-1}(-B\vec{u}_s) + \vec{x_f} - \vec{x_s}$. After the linearization, we get $\vec{x'}(k)$ and then obtain the exact states $\vec{x}(k) = \vec{x'}(k) + x_s - (A - I)^{-1}(-B\vec{u}_s)$.

Here is an example of minimal energy (control cost) solution of linear discrete-time system $f = A\vec{x'}(k) + B\vec{u}(k)$, with fixed final time step τ, initial state $\vec{x_0}$, final state $\vec{x_f}$ and objective function

$$J_\epsilon = \sum_{k=0}^{\tau} \vec{u}^\intercal(k)\vec{u}(k). \quad [14]$$

After applying Lagrangian multipliers $\vec{\lambda}$, we get

$$\begin{aligned} \vec{\lambda}(k) &= A^\intercal \vec{\lambda}(k+1), && k \in [0, \tau - 1], \tau \text{ equations}, \\ 0 &= \vec{u}(k) + B^\intercal \vec{\lambda}(k+1), && k \in [0, \tau - 1], \tau \text{ equations}, \\ \vec{x'}(k+1) &= A\vec{x'}(k) + B\vec{u}(k), && k \in [0, \tau - 1], \tau \text{ equations}, \\ \vec{x'}(0) &= \vec{x'_0}, && 1 \text{ equation}, \\ \vec{x'}(\tau) &= \vec{x'_f}, && 1 \text{ equation}. \end{aligned} \quad [15]$$

The variables are $X' = [\vec{x'}(0), \dots, \vec{x'}(\tau)]$, $U = [\vec{u}(0), \dots, \vec{u}(\tau - 1)]$, $\Lambda = [\vec{\lambda}(0), \dots, \vec{\lambda}(\tau)]$. Total number of vector variables and functions are both $3\tau + 2$. Let $A_{B;\tau} = [A^{\tau-1}B, \dots, A^0 B]^\intercal$, when $\text{rank}(A_{B;\tau}) = N$, The solution of Eq. (15) is

$$\begin{aligned} \vec{\lambda}(\tau) &= (A_{B;\tau}^\intercal A_{B;\tau})^{-1}(A^\tau \vec{x'_0} - \vec{x'_f}), \\ \vec{\lambda}(k) &= (A^{\tau-k})^\intercal \vec{\lambda}(\tau), \\ \vec{u}(k) &= -B^\intercal \vec{\lambda}(k+1), \\ \vec{x}(k) &= A^k \vec{x'_0} - (A_{B;k}^\intercal A_{B;k})\vec{\lambda}(k). \end{aligned} \quad [16]$$

The state trajectories with optimal energy control are reported in Supplementary Fig. S4 and Fig. S5. The state trajectories of the linearized approximated system is extremely close to the solution in the actual nonlinear system (Supplementary Fig. S4), while the linearized system needs by orders of magnitude less computational time but around 30% more energy (Fig. S3 and Supplementary Fig. S5). We also applied optimal control in continuous systems in global risk network, the energy of any continuous system is only slightly (around 1%) smaller than that of the corresponding discrete system (Fig. S3). Therefore, we only discuss the discrete system in this work.

Linear Quadratic Regulator

Furthermore, we may also need all the intermediate states to be as close to zero as possible, we use Linear-Quadratic Regulator (LQR) with the objective function to minimize total cost

$$J_t = \vec{x}^\intercal(\tau)Q_f\vec{x}(\tau) + \sum_{k=0}^{\tau-1}[\vec{x}^\intercal(k)Q\vec{x}(k) + \vec{u}^\intercal(k)R\vec{u}(k)]. \quad [17]$$

The advantage of LQR objective function is to find the best control strategy that minimizes the costs in terms of both control inputs and all the intermediate states. It is meaningful in many scenarios. For example, in global risk network control, keeping the activities of risk at low level improves the safety of the world during the entire control process.

Given finite control time steps $k = \tau, \tau - 1, \ldots, 1$, the optimal solution for Eq. (17) is from backward Riccati recursion,

$$\begin{aligned} P(k-1) &= Q + A^\intercal P(k)A - A^\intercal P(k)B[R + B^\intercal P(k)B]^{-1}B^\intercal P(k)A, \\ K(k-1) &= -[R + B^\intercal P(k)B]^{-1}B^\intercal P(k)A, \\ \vec{u}(k-1) &= K(k-1)\vec{x}(k-1), \\ \vec{x}(k) &= [A + BK(k-1)]\vec{x}(k-1), \end{aligned} \quad [18]$$

where $P(\tau) = Q_f$.

As we mentioned above, in our linearized CARP model, the state update function is $\Delta\vec{x}(k+1) = A\Delta\vec{x}(k) + B\Delta\vec{u}(k)$. Thus, we can not directly apply LQR solution in Eq. (18). Given $\vec{x}^o = \vec{0}$ and $\vec{u}^o = \vec{0}$ as state $\vec{x}$ and control input $\vec{u}$ target sequences, we have references $\Delta\vec{x}^o = \vec{x}^o - \vec{x}_s$ and $\Delta\vec{u}^o = \vec{u}^o - \vec{u}_s$ for $\Delta\vec{x}$ and $\Delta\vec{u}$. Thus, the formulation of discrete-time optimal control problems with reference tracking is to minimize

$$\begin{aligned} J_{t'} = \; & [\Delta\vec{x}(\tau) - \Delta\vec{x}^o]^\intercal Q_f[\Delta\vec{x}(\tau) - \Delta\vec{x}^o] \\ & + \textstyle\sum_{k=0}^{\tau-1}\{[\Delta\vec{x}(k) - \Delta\vec{x}^o]^\intercal Q[\Delta\vec{x}(k) - \Delta\vec{x}^o] \\ & + [\Delta\vec{u}(k) - \Delta\vec{u}^o]^\intercal R[\Delta\vec{u}(k) - \Delta\vec{u}^o]\}, \end{aligned} \quad [19]$$

subject to $\Delta\vec{x}(k+1) = A\Delta\vec{x}(k) + B\Delta\vec{u}(k)$ and $\Delta\vec{x}(0) = \vec{x}_0 - \vec{x}_s$. It can be firstly solved backward from $k = \tau - 1$ to $k = 0$, with $P(\tau) \equiv Q_f$ and $\vec{s}(\tau) \equiv -Q_f\Delta\vec{x}^o$:

$$\begin{aligned} M(k) &= [I + BR^{-1}B^\intercal P(k+1)]^{-1}, \\ P(k) &= Q + A^\intercal P(k+1)M(k)A, \\ \vec{s}(k) &= A^\intercal[I - P(k+1)M(k)BR^{-1}B^\intercal]\vec{s}(k+1), \\ & \quad + A^\intercal P(k+1)M(k)B\Delta\vec{u}^o - Q\Delta\vec{x}^o, \end{aligned} \quad [20]$$

then forward from $k = 0$ to $k = \tau - 1$, with $\Delta\vec{x}(0) = \vec{x}(0) - \vec{x}_s$:

$$\begin{aligned} \vec{v}(k) &= M(k)B[\Delta\vec{u}^o - R^{-1}B^\intercal\vec{s}(k+1)], \\ \Delta\vec{x}(k+1) &= M(k)A\Delta\vec{x}(k) + \vec{v}(k), \\ \vec{\lambda}(k+1) &= P(k+1)\Delta\vec{x}(k+1) + \vec{s}(k+1), \\ \Delta\vec{u}(k) &= \Delta\vec{u}^o(k) - R^{-1}B^\intercal\vec{\lambda}(k+1). \end{aligned} \quad [21]$$

The detailed solution for LQR with reference tracking can be found in Ref. (5).

We compare two optimizers of optimal energy control and LQR control (Supplementary Fig. S2), and note that the final state is strictly controlled to the desired state $\vec{0}$ in optimal energy control; while in LQR control, the states are close to but not exactly zero. Optimal energy control has a smaller energy ($\vec{u}^\intercal\vec{u}$), while LQR control has a smaller total cost ($\vec{x}^\intercal Q\vec{x} + \vec{u}^\intercal R\vec{u}$). We also include LQR with different $Q_f = Q$ and R matrices, a higher Q leads to a tighter state constraint, while a higher R leads to a looser state constraint.

Supplementary Figures

References

1. 8. World Economic Forum Global Risks Report. https://www.circleofblue.org/wp-content/uploads/2015/01/WEF_Global_Risk_Report2006.pdf, 2006. Accessed: 2019-09-10.
2. Boleslaw K Szymanski, Xin Lin, Andrea Asztalos, and Sameet Sreenivasan. Failure dynamics of the global risk network. *Scientific reports*, 5(10998), 2015.
3. Zoran Gajic. *Linear dynamic systems and signals.* Prentice Hall/Pearson Education, 2003.
4. Andrew P Sage and Chelsea C White. *Optimum systems control.* Prentice Hall, 1977.
5. Athanasios Sideris and James E Bobrow. An efficient sequential linear quadratic algorithm for solving nonlinear optimal control problems. In *Proceedings of the 2005, American Control Conference, 2005.*, pages 2275–2280. IEEE, 2005.

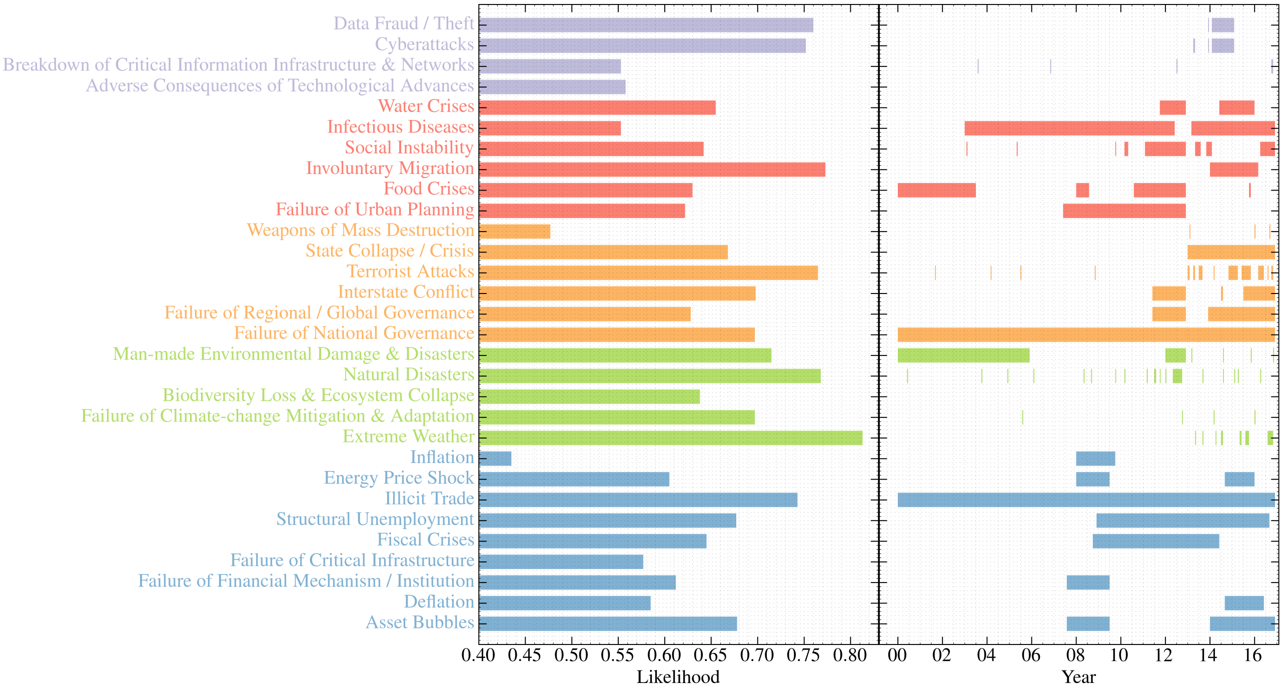

Fig. S1. Normalized likelihoods and historical events data points from 2000 Jan to 2016 Dec for each risk in 2017 global risk network in five categories (with five different colors). The likelihood before normalization is in a 1-7 scale. After normalization with $L_i = \frac{l_i - 1}{7-1}$, the values are within $[0, 1]$. The historical events data points are collected by human knowledge from online resources. A colored bar represents a detected active risk-event.

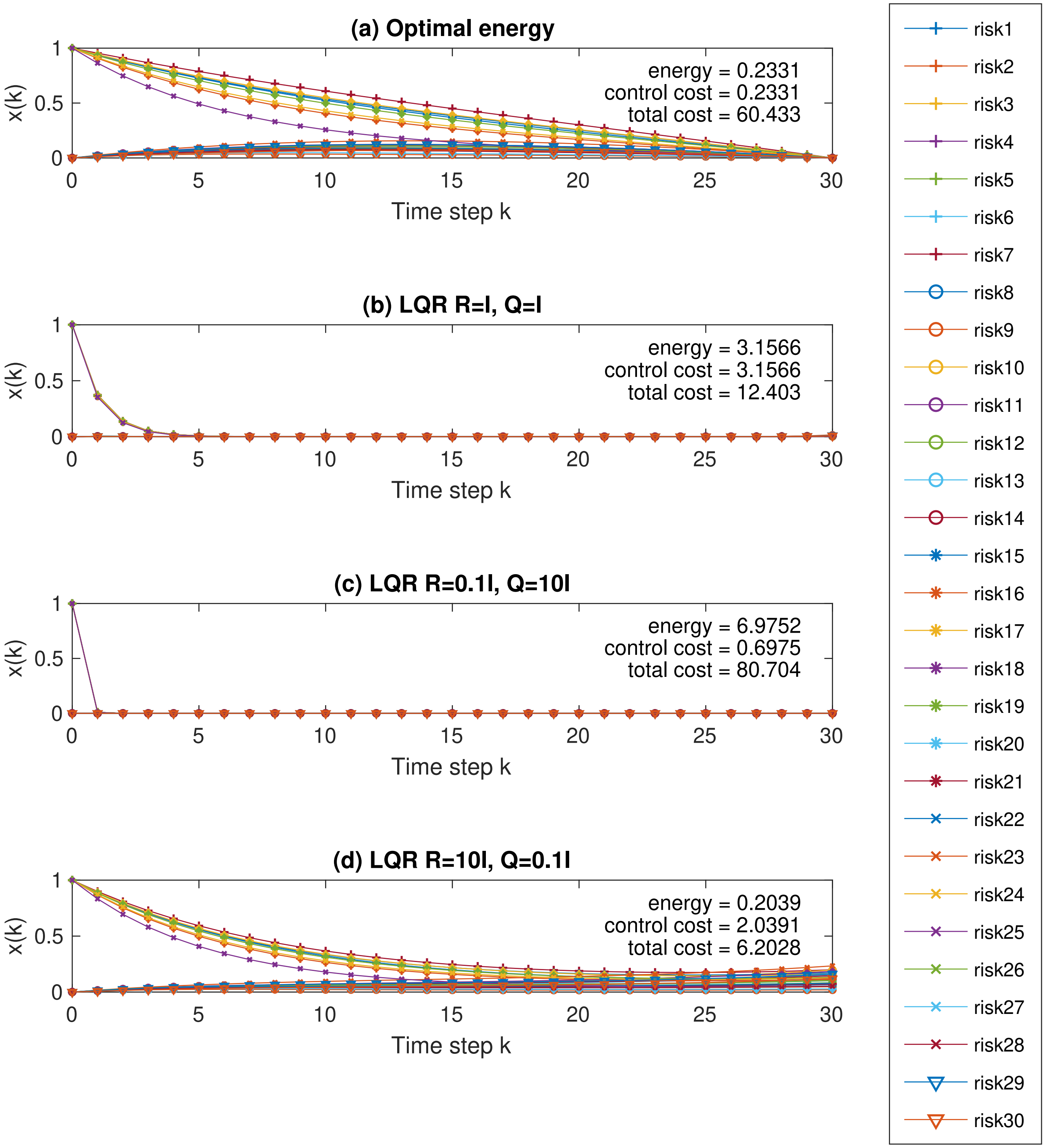

Fig. S2. Comparison of different optimizers. (a). Optimal energy control with final state constraint $\vec{x}_f = \vec{0}$. (b). LQR control with $R = I$, $Q = I$. (c). LQR control with smaller R and bigger Q. (d). LQR control with bigger R and smaller Q. Energy is $\sum_{k=0}^{\tau-1} \vec{u}^{\intercal}(k)\vec{u}(k)$. Control cost is $\sum_{k=0}^{\tau-1} \vec{u}^{\intercal}(k)R\vec{u}(k)$. Total cost is $\vec{x}^{\intercal}(\tau)Q\vec{x}(\tau) + \sum_{k=0}^{\tau-1} \vec{x}^{\intercal}(k)Q\vec{x}(k) + \vec{u}^{\intercal}(k)R\vec{u}(k)$.

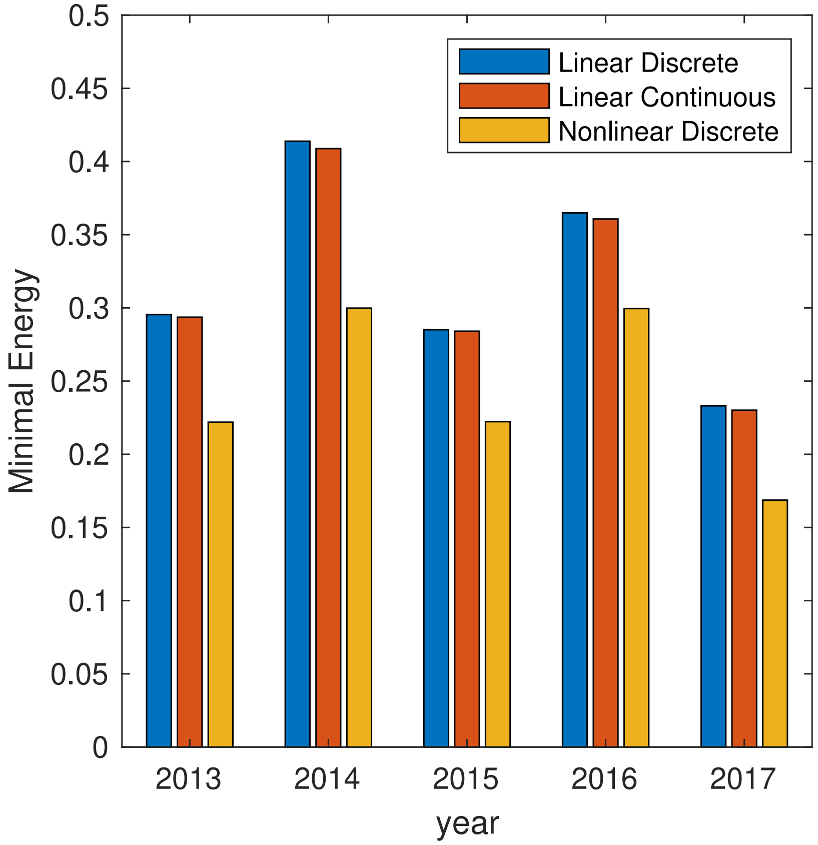

Fig. S3. **The optimal energy of five global risk networks with linear discrete, linear continuous and nonlinear discrete control.** The energy $\sum_{k=0}^{\tau-1} \vec{u}^{\intercal}(k)\vec{u}(k)$ is summated over $\tau = N$ control time steps.

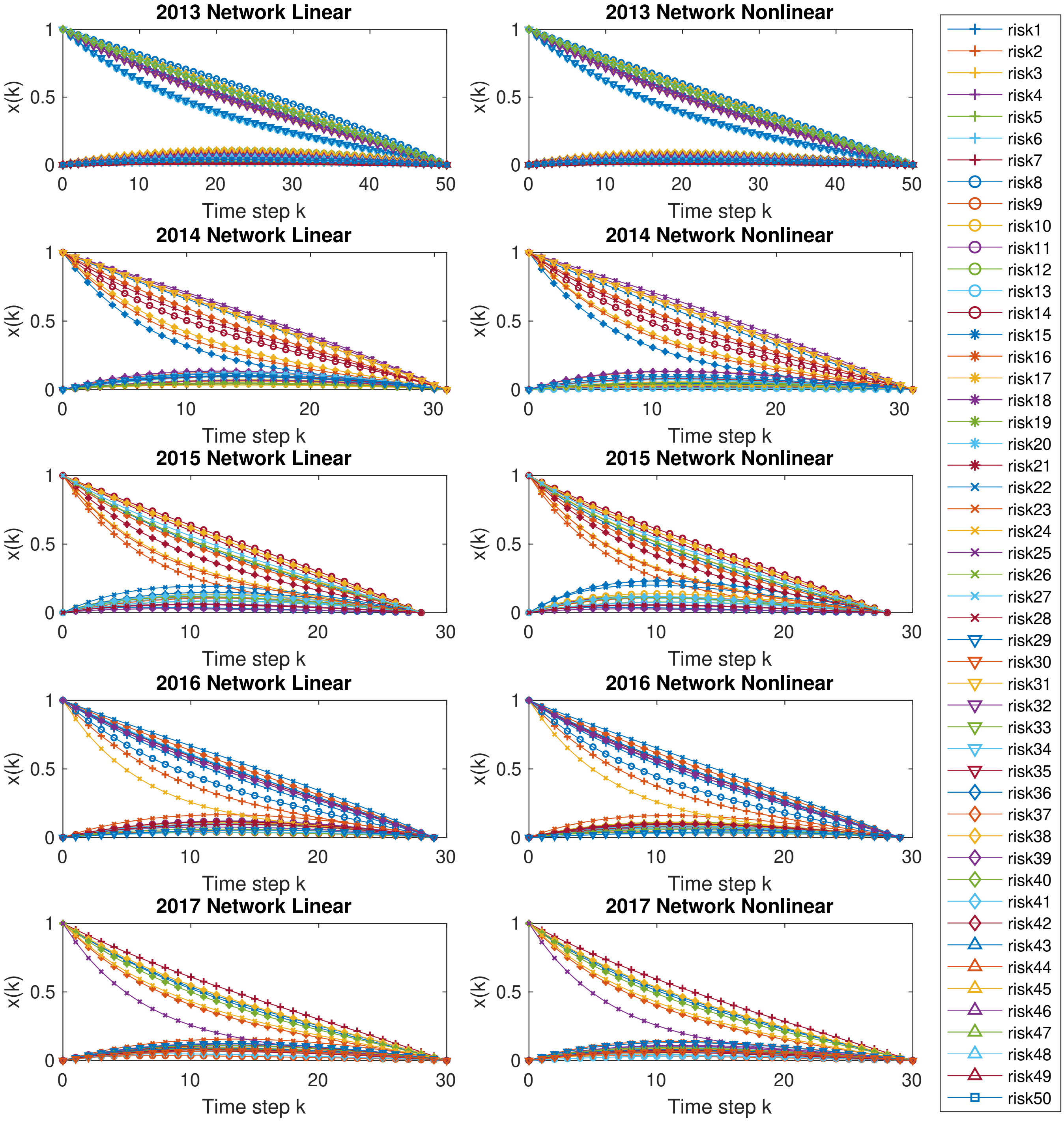

Fig. S4. State trajectories with optimal energy control of linearized and nonlinear global risk networks from 2013 to 2017.

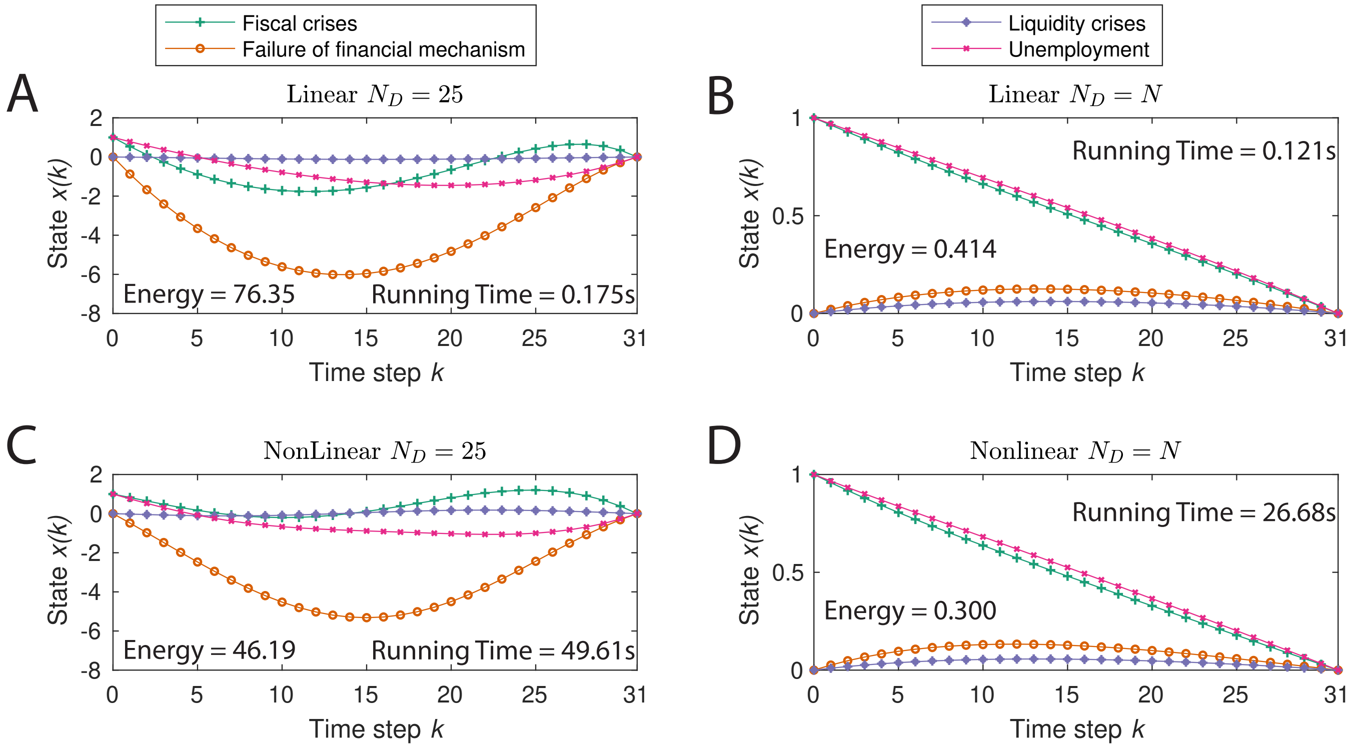

Fig. S5. Comparison of the states, energy and computational time of linear and nonlinear control of 2014 global risk network. A. and C. The states trajectories of risks with discrete optimal control with negative intermediate states (N_D = 25) at each time step k. B. and D. The states trajectories of risks with discrete non-negative optimal control ($N_D = N$) at each time step k. A. and B. The results of linear approximation. C. and D. The results of nonlinear numerical solution.
